# Relationship of erithrocyte membrane polyunsaturated fatty acid composition with biochemical hypogonadism in aging male: evidence from a cross-sectional analysis

**DOI:** 10.1007/s00345-026-06438-6

**Published:** 2026-05-13

**Authors:** Taha Ucar, Saifullah Khan, Ates Kadioglu

**Affiliations:** 1 Independent Researcher, İstanbul, Turkey; 2 İstanbul Faculty of Medicine, Department of Urology, Division of Andrology, İstanbul University, Çapa, 34390 İstanbul, Turkey; 3 İstanbul Faculty of Medicine, Department of Urology, İstanbul University, İstanbul, Turkey

**Keywords:** Hypogonadism, Testosterone, Fatty acids, Arachidonic acid, Erythrocytes, Membrane lipids, Cross-sectional studies

## Abstract

**Purpose:**

To investigate the relationship between polyunsaturated fatty acids and biochemical hypogonadism in aging males with a cross-sectional NHANES data.

**Methods:**

A total of 1165 men aged ≥ 65 years old, who had complete data on total testosterone levels and erythrocyte membrane fatty acid composition measurements, were included from the National Health and Nutrition Examination Survey (NHANES) 2021–2023 dataset. Biochemical hypogonadism was defined as total testosterone ≤ 300 ng/dL. Age, body mass index (BMI), waist circumference, diabetes mellitus (DM), fatty acid enzyme activities, HDL, LDL, triglycerides, and total cholesterol were also recorded.

**Results:**

A total of 1,165 men were analyzed, of whom 859 had complete total testosterone data. In multivariate logistic regression analysis using continuous variables, higher arachidonic acid levels were independently associated with lower odds of biochemical hypogonadism (OR 0.80, 95% CI 0.66–0.97, *p* = 0.026). Triglyceride levels (OR 1.005, 95% CI 1.001–1.009, *p* = 0.006) and waist circumference (OR 1.037, 95% CI 1.019–1.056, *p* < 0.001) were positively associated with biochemical hypogonadism. Diabetes mellitus showed a borderline association with hypogonadism but did not reach statistical significance after adjustment (OR 1.54, 95% CI 0.93–2.56, *p* = 0.09). HDL cholesterol, palmitoleic acid, docosahexaenoic acid, and linoleic acid were associated with biochemical hypogonadism in univariate analysis but did not remain significant after multivariate adjustment.

**Conclusion:**

Arachidonic acid (AA), a component of the omega-6 fatty acid pathway, levels were independently associated with lower odds of biochemical hypogonadism. whereas waist circumference, diabetes mellitus and triglycerides were associated with biochemical hypogonadism.

## Introduction

Late-onset hypogonadism is a common endocrine disorder with specific symptoms in aging men and is increasingly recognized as a metabolic condition. The prevalence of symptomatic male testosterone deficiency ranges between 2.1% and 11.8% [1, 2]. Large-scale studies in the general population have repeatedly shown that conditions like obesity, excess belly fat, and diabetes are key factors that lower testosterone levels. These effects occur through processes such as insulin resistance, ongoing inflammation, increased conversion of testosterone to estrogen, and disruptions in the normal functioning of the hormonal system that controls testosterone production [3–5].

Despite similar metabolic profiles, considerable individual variability in testosterone concentrations occurs in aging males, indicating that additional biological factors may contribute to androgen deficiency [6]. Polyunsaturated fatty acid (PUFA) metabolism may represent a susceptible and underexplored determinant of steroidogenesis. In addition to cholesterol availability, fatty acids influence membrane fluidity, mitochondrial function, intracellular signaling, and steroidogenic enzyme activity [7, 8].

There is a lack of evidence regarding the effect of PUFAs on testosterone metabolism. This study evaluates the relationship between PUFAs, lipid metabolism enzyme activity, and biochemical component of late onset hypogonadism with the Erythrocyte (RBC) membrane fatty acid composition technique. Blood fatty acid compositions are an indicator of long-term lipid metabolism. Since erythrocytes have a mean life span of 120 days, blood fatty acid composition relying on erythrocytes indicates the long-term fatty acid levels rather than the plasma levels of fatty acids [9]. Data were gathered from the National Health and Nutrition Examination Survey (NHANES), a cross-sectional survey providing data from the noninstitutionalized US population.

## Materials and methods

### Study population

Men aged ≥ 65 years with available total testosterone, RBC fatty acid profiles, BMI, waist circumference, and diabetes status were included. Participants with missing data for any of these variables were excluded [2]. A total of 859 men with available total testosterone data were included from the initial cohort of 1165 men. Biochemical hypogonadism was defined as total testosterone ≤ 300 ng/dL, consistent with recommendations from the Endocrine Society and American Urological Association [10, 11].

### Study endpoints and assessment

Total RBC membrane fatty acid composition was assessed. Individual fatty acids were analyzed separately, including omega-6 polyunsaturated fatty acids arachidonic acid (20:4 n-6), linoleic acid (18:2 n-6), γ-linolenic acid (18:3 n-6), and dihomo-γ-linolenic acid (20:3 n-6); omega-3 polyunsaturated fatty acids α-linolenic acid (18:3 n-3), eicosapentaenoic acid (20:5 n-3), docosapentaenoic acid (22:5 n-3), and docosahexaenoic acid (22:6 n-3); as well as major saturated fatty acids including myristic acid (14:0), palmitic acid (16:0), stearic acid (18:0), and arachidic acid (20:0), and major monounsaturated fatty acids including palmitoleic acid (16:1 n-7), oleic acid (18:1 n-9), and vaccenic acid (18:1 n-7).

The primary endpoint of the study was to identify the relationship between PUFAs and biochemical hypogonadism. Secondarily, the study aims to identify the related factors of biochemical hypogonadism in a detailed model, including metabolic syndrome parameters and PUFAs.

### Statistical analysis

Mann-Whitney U and chi-square tests were used where appropriate. Variables that were significant in univariate analysis were included in the multivariate logistic regression model. Multicollinearity among independent variables was assessed using variance inflation factors (VIF). Since BMI and waist circumference were strongly correlated, BMI was excluded from the final model. After exclusion of BMI, no relevant multicollinearity was observed among the remaining variables. Odds ratios(OR) with 95% confidence intervals were also reported.

## Results

A total of 1,165 men aged 65 and older were analyzed, and 859 men with complete testosterone data were included. Biochemical hypogonadism criteria (total testosterone ≤ 300 ng/dL) were met in 209 (24.3%) men. Age was comparable between the two groups. Those diagnosed with hypogonadism had a substantially higher metabolic burden, demonstrated by increased BMI, lower HDL, greater waist circumference, and a notably higher rate of diabetes mellitus in comparison to men with normal testosterone levels. (Table 1). A multivariate model was also composed with clinically significant variables.

According to univariate analysis, arachidonic acid (AA) levels were significantly lower in men with biochemical hypogonadism compared to non-hypogonadal men (9.09 vs. 9.54, *p* < 0.001). Palmitoleic acid (POA) levels were significantly higher in hypogonadal men (0.257 vs. 0.247, *p* = 0.003). Linoleic acid levels were also higher in hypogonadal men (0.054 vs. 0.049, *p* < 0.001). Similarly, DHA levels were slightly higher in hypogonadal men (14.2 vs. 14.0, *p* = 0.010) (Table 1).

**Table 1:** Clinical variables and RBC membrane fatty acids according to hypogonadism status

Variable	Hypogonadal median	*n*	Non-hypogonadal median	*n*	*p*
Waist circumference (cm)	107	206	101	646	<0.001*
BMI (kg/m²)	30.1	207	28.2	645	<0.001
Triglyceride (mg/dL)	151	208	132	649	<0.001*
HDL cholesterol (mg/dL)	47	208	50	649	<0.001
Diabetes mellitus n (%)	83 (39.7%)	209	127 (19.5%)	650	<0.001
Age (years)	70	209	69	650	0.226
Total cholesterol (mg/dL)	192	208	195	649	0.287
LDL cholesterol (mg/dL)	115	208	118	649	0.288
Fatty acids					
Arachidonic acid (20:4 n-6)	9.09	208	9.54	649	<0.001*
Linoleic acid (18:2 n-6)	0.054	208	0.049	649	<0.001
Docosahexaenoic acid (22:6 n-3)	14.2	209	14.0	648	0.010
Palmitoleic acid (16:1 n-7)	0.257	206	0.247	640	0.003
DGLA (20:3 n-6)	1.62	208	1.55	649	0.145
γ-linolenic acid (18:3 n-6)	0.021	208	0.020	649	0.403
Oleic acid (18:1 n-9)	17.5	209	17.4	650	0.728
Palmitic acid (16:0)	21.7	208	21.4	649	0.088
Stearic acid (18:0)	20.5	209	20.4	650	0.703
Myristic acid (14:0)	0.82	206	0.82	640	0.974
Arachidic acid (20:0)	0.43	208	0.42	649	0.080
Eicosenoic acid	0.31	206	0.30	640	0.158
Eicosadienoic acid	0.21	206	0.21	640	0.765
Pentadecanoic acid	0.34	206	0.33	640	0.443

*Independently associated with hypogonadism in multivariate logistic regression analysis

Regarding the multivariate logistic regression analysis constructed after multicollinearity assessment, waist circumference (OR 1.037, 95% CI 1.019–1.056, *p* < 0.001) and triglyceride levels (OR 1.005, 95% CI 1.001–1.009, *p* = 0.006) were independently associated with hypogonadism. In contrast, arachidonic acid was inversely associated with hypogonadism when modeled as a continuous variable (OR 0.804 per 1% increase, 95% CI 0.664–0.974, *p* = 0.026). HDL cholesterol, palmitoleic acid, linoleic acid, and DHA were not independently associated with hypogonadism in the adjusted model. The logistic regression model demonstrated adequate calibration according to the Hosmer–Lemeshow test (χ² = 5.60, df = 8, *p* = 0.69).

## Discussion

This study reveals that biochemical hypogonadism in men aged 65 years and older is associated with AA, DM, waist circumference and TG according to multivariate logistic regression analysis. DM, waist circumference and TG were independently associated with biochemical hypogonadism, while AA was independently associated with lower odds of biochemical hypogonadism.

AA is an important structural component of cell membranes and is a precursor of eicosanoids involved in intracellular signalling pathways, steroid hormone synthesis, and mitochondrial activity [12]. Experimental studies have suggested that membrane lipid composition may influence steroidogenesis and mitochondrial function in Leydig cells. Fatty acids may contribute to cholesterol transport and StAR protein activity, which are important steps of testorone synthesis. This unproven mechanism may partly explain the observed relationship between arachidonic acid and testosterone. However, most of the available evidence is derived from experimental models, and the underlying biological mechanisms remain incompletely understood [12].

Emerging evidence suggests that the relationship between test osterone and metabolic parameters may reflect reverse causality. Obesity, insulin resistance and hypertriglyceridemia may supress hipothalamic-pituitary- gonadal axis to reduce testosterone levels as well as low testosterone levels can promote increased adiposity and worsening metabolic profile. Increased visceral adiposity is related with aromatase activity that converts testosterone to estradiol in peripheral tissues. Therefore metabolic syndrome and hypogonadism appear to form a vicious cycle affecting both metabolic and hormonal pathwaysi making it diffucult to define a one-way relationship with testosteron and metabic syndrome and fatty acids [13, 14]. Waist circumference was preferred over BMI due to its high relevance with visceral adiposity and association with insulin resistence and inflamation. Previous studies have revealed that waist circumference correletes with testosterone levels better than BMI regarding as a predictor for hypogonadism [15, 16].

The Following limitations of the present study should be acknowledged. Since the NHANES dataset is cross-sectional, it cannot establish causality or rule out reverse causation. Second, even with the wide range of covariates included, the personal choices and environmental factors cannot be ruled out. In addition due to inherent characteristics of NHANES dataset, the definition and diagnosis of hypogonadism or late onset hypogonadism was only based on a single Testosterone measurements without assessment of hypogonadism-related symptoms. Survey weights were not applied despite complex sampling design of NHANES. Therefore, the results may not be fully generalizable to the US population. Another limitation is the lack of additional endocrine parameters such as SHBG, LH, estradiol, insulin, and CRP, as well as the absence of information on potential confounders affecting, chronic diseases, testosterone levels, including testosterone therapy, 5α-reductase inhibitors, and GnRH analogues. Furthermore, although erythrocyte fatty acid composition is one of the best markers for assessing long-term lipid metabolism, it remains inefficient for detecting fatty acid levels in testicular tissue and the specific microenvironment associated with steroid hormone production.

In conclusion, this study suggests that arachidonic acid, an omega-6 fatty acid, may be inversely associated with biochemical hypogonadism in older men, while waist circumference, TG were independently associated with higher odds for biochemical hypogonadism. These findings suggest that fatty acid metabolism may represent a relevant component of testosterone regulation beyond conventional metabolic parameters such as HDL cholesterol, LDL cholesterol, and diabetes mellitus and warrant further investigation in longitudinal studies.

## Data Availability

The data used in this study are publicly available from the National Health and Nutrition Examination Survey (NHANES), conducted by the National Center for Health Statistics (NCHS), Centers for Disease Control and Prevention (CDC). NHANES datasets can be accessed at: https://www.cdc.gov/nchs/nhanes/and downloaded from the NHANES data repository at: https://wwwn.cdc.gov/nchs/nhanes/”.
